# The Book World of Medicine and Science

**Published:** 1894-03-31

**Authors:** 


### THE BOOK WORLD OF MEDICINE AND SCIENCE.

ATLAS OF CLINICAL MEDICINE. By Dr. BYROM BRAMWELL.

IN this part of his valuable Atlas, Dr. Byrom Bramwell gives a full account of exophthalmic goitre, of acromegaly, and of the dermatitis that occurred as an epidemic in 1891 in the Marylebone and Paddington Poor Law Infirmaries. There is also the report of a case of unilateral hypertrophy of the face sent to Dr. Bramwell by Dr. Montgomery, Professor of Diseases of the Skin at the University of California. The admirable illustrations which accompany the above are of course the main feature. Mr. Williamson is an able draughtsman, and in addition to those illustrations mentioned in the text, an excellent drawing of an old woman has been added to show the superficial characteristics of the skin of the face in old age. While, however, the book is called an atlas, all that is known of the diseases illustrated is summarised in an interesting way. In his article upon exophthalmic goitre when dealing with prognosis Dr. Bramwell states that probably in one-third or one-quarter of the patients affected the symptoms subside and a complete cure is established. In other cases the disease improves up to a certain point, after which it remains stationary, some or all of the symptoms persisting in a less severe form. In such cases there may be a relapse. In a small proportion of cases there is an acute onset, followed after a time by sudden disappearance, while in a fourth class the disease progresses from bad to worse until death takes place by asthenia, dropsy due to cardiac valvular incompetence, or by lung complications. In any individual case it is difficult to give a definite opinion as to the probable issue. Dr. Bramwell thinks that more attention must be paid to general nervousness and to frequency of the pulse than to marked enlargement of the thyroid or prominence of the eyes. Of the curious disease acromegaly, Dr. Bramwell has had two cases under his care, and remembers two others, the peculiar features of which he noticed before the disease was described. Several photographs are given illustrating his cases. In the group of photographs taken from one of these, the change of expression of the face produced by the lengthening of the jaw is well shown. A bright, intelligent-looking girl becomes in a few years a woman of sad lethargic appearance. The thickening of the softer tissues of the extremities is illustrated by photographs of the soles of the feet, and outline drawings are given, showing the actual size of the hands in one case. Of the dermatitis described by Dr. Savill, two coloured illustrations are given, one representing the dry, the other the moist variety. In addition to illustrations of both sides of the face in the case of unilateral hypertrophy, their is one of a cast of the roof of the mouth showing the enormous thickening of the bone on the side affected.

NORMAL LABOUR. By MOFFAT FLYNN, F.R.C.S., Leicester. (*Provincial Medical Journal* Office, 1894.)

This little pamphlet of eight pages gives a sketch of the method adopted in the Frauen Klinik in Berlin for the management of normal labour, in the cases under the care of Professor Olshausen.

The chief peculiarities of the method are the care taken in external antisepsis, the avoidance of vaginal examination, and the management of the third stage.

"The woman after she starts in labour has a bath at a temperature of 28 deg. C. of soap and water, after which she has a soap-and-water enema; she is then placed on her back in bed, and the vulva is well washed with warm water and soap; this is done with juta; no sponge is ever used; next, corrosive sublimate, 1 in 1,000, is poured over the external genitals."

The pelvis is then measured, the water drawn off, and the diagnosis of the position of the child made. After the position is ascertained, if it be normal, no further internal examination is made unless it be absolutely necessary. There seems, in fact, to be a tendency towards omitting vaginal examinations altogether in some of the Berlin hospitals, the position and course of labour being made out by abdominal palpation.

The doctors and the nurses render their hands and arms aseptic as follows: First they are washed in warm water and soap, a brush being used; they are then carefully dried in a towel. This is of course to prevent any soap being carried into the corrosive sublimate solution which it would decompose. Next they are put for three minutes into 1 in 1,000 corrosive sublimate solution, then in alcohol (80 per cent.) for one minute, and lastly dipped in the corrosive sublimate solution, wet out of which they go to any vaginal examination.

The 1 in 1,000 corrosive sublimate solution is also used for cleansing the external genitals, but it is not used for douching.

In case of intra-uterine manipulations, putrescent endometritis, or other causes requiring a douche, a 5 per cent. solution of carbolic acid is employed, but after normal labour, or simple forceps cases, no douche is used. As to the management of the third stage, as soon as the child is born the mother is turned on her back, but no hand is laid on the abdomen and no drugs are given. In about twelve minutes the placenta is expressed, but after this there is no further pressure, and no binder is put on.

Mr. Flynn adds that although some high authorities favour vaginal injections of 2 per cent. carbolic acid, this has of late been going out of fashion, and recommends creolin 2 per cent. where douching is required; he also dislikes the let-alone method of dealing with the patient afterwards, preferring to give a dose of ergot as soon as the placenta is expressed, and to apply a binder.

For those who wish for information on the Berlin antiseptic method in midwifery these few pages will be useful. At the same time it must not be forgotten that antisepsis quite as complete, and in some ways even more thorough, is now insisted on in the London hospitals, and that, although the books are somewhat indefinite on the subiect, the teaching in some of the London schools is very positive in regard of the absolute necessity for a most careful application of antiseptic methods to obstetrics.

THE OXFORD MUSEUM. By HENRY W. ACLAND, M.D., and JOHN RUSKIN, M.A. (George Allan, London and Orpington. 1893.)

"Thirty-four years have elapsed since the few pages which follow have been out of print in their present form," the author informs us in his preface to the reprint of this interesting little volume. Of late years Sir Henry Acland has been frequently pressed to re-issue it. This he now has done, together with two letters from Mr. Ruskin, whose interest in the building of the Oxford Museum is of a deep and a personal order. To the much venerated art critic is due in a great measure the beauty of its internal decoration, as to Dr. Acland its actual foundation. After dealing in broad terms with the state of science and art in the middle of the century, the author initiates us into the many and various functions of the Museum, and the work it was designed to effect—a work which has since made itself well known and felt by all interested and engaged in science. Less well known is the actual history of the building itself, and the difficulties which beset the path of those energetic constructors who evolved from the wilds of an "unploughed field" this museum which boasts itself by no means the least conspicuous among the University's solid acquisitions. Speaking of his project in the early days of its planning, and when the serious question of funds was being raised, Sir Henry Acland writes: "Evary grant was carried in Convocation by a narrow majority. That for the gaspipes, for instance, was carried; that for the burners was lost!" We learn much in the course of this little volume of the changes brought about in a University education, and in the expansion of scientific teaching since the days when its furtherance met with such strenuous opposition, and before the Oxford Museum asserted its function in the able manner for which it has since been remarkable. Sir Henry Acland tells its story in a delightfully historic and narrative manner.

